# Confronting the Neurocognitive Toll of Sepsis Survivors

**DOI:** 10.14789/ejmj.JMJ25-0046-P

**Published:** 2025-12-18

**Authors:** ZAINAB SALAHUDDIN

**Affiliations:** 1 Department of Clinical Psychology, SZABIST University, Islamabad, Pakistan; 1 Department of Clinical Psychology, SZABIST University, Islamabad, Pakistan

**Keywords:** sepsis, post-intensive care syndrome, cognitive impairment, delirium, survivorship

## Abstract

Cognitive impairments tend to persist among survivors of sepsis long after discharge, and may involve memory, attention, and executive function. These deficits are driven by chronic sequelae of neuroinflammation, blood-brain barrier breakdown, and an extended period of delirium in critical illness. Although post-intensive care syndrome is increasingly acknowledged, cognitive recovery is not systematically assessed during follow-up. This Perspective emphasises the crucial importance of early recognition of cognitive impairment, the use of multidisciplinary rehabilitation, and the incorporation of screening for cognitive dysfunction in post-ICU care to ensure that sepsis care becomes concerned not only with survival but also with the restoration of neurocognitive well-being.

Sepsis has become a significant target, not just for its immediate mortality but increasingly for what comes after: the long and often hidden road to recovery. The recent paper on *Neurocognitive Outcomes After Critical Illness: Post-ICU Syndrome in Sepsis Survivors* brings long-needed attention to one of the most hidden but impactful outcomes of survival, long-term cognitive dysfunction. As we continue to answer the call and lower mortality from sepsis and critical illness, it is time for us to turn some of our focus from saving lives to healing minds.

Sepsis may induce long-term cerebral dysfunction in survivors, resulting in lasting deficits across cognitive domains (including memory, attention and executive function). These deficits arise through various mechanisms, including blood-brain barrier disruption, neuroinflammation, and the presence of amyloid β and tau proteins, which can be severe and manifest in both structural and functional brain changes^1)^. For instance, in an extensive population-based study utilising the Health and Retirement Study, survivors with severe sepsis were 3.3 times more likely to have moderate to severe cognitive impairment compared with subjects hospitalised for non-sepsis reasons, with effects persisting for at least 8 years^2)^. These deficits are not just numbers in neuropsychological tests; instead, they translate into a lack of independence, weakened productivity in the workplace, diminished quality of life and increased burden for caregivers and health care systems^3)^.

Sepsis-associated brain injury is multifactorial. Acute events, sepsis-associated encephalopathy (SAE), delirium, hypoxemia, and hypotension are prone to long-term injury^4)^. Older age, length of stay in ICU, mechanical ventilation and cognitive frailty before illness are the leading risk factors^5)^.

Working memory and visual attention are selectively impaired and associated with executive dysfunction^6)^. A prospective cohort study found that longer delirium duration in the ICU was independently associated with long-term cognitive impairment at 3 and 12 months post-discharge after adjusting for confounders. This underscores delirium as a potentially treatable risk factor for long-term cognitive decline in survivors of ICU^7)^. The term "post-intensive care syndrome" (PICS), which includes physical, mental and cognitive disorders, reflects the complex pathways, from inflammation to mitochondrial dysfunction, responsible for long-term sequelae among ICU survivors^8)^.

Although evidence continues to accumulate, post-ICU cognitive outcomes are still under-evaluated as part of the standard clinical care. In most health systems, and particularly in low- and middle- income countries, the emphasis of post-CC follow- up remains predominantly physical, respiratory, renal or mobility outcomes, with little attention to cognitive and psychological health. Established standards for formal neuropsychological evaluations and tests are often unattainable, as individuals may struggle to express or fail to describe their cognitive symptoms due to the absence of clear neurological signs. That leaves a vast number of patients whose problems never get acknowledged and addressed. A multidisciplinary approach, including cognitive screening, psychological assessment and rehabilitation activities, should be included in post-ICU care programs^9)^.

Interventions that could decrease the neurocognitive load have to start in the ICU. Avoidance or attenuation of delirium through early rehabilitation, careful sedation strategies, and a supportive environment is beneficial in the long term. Lighter sedation regimens, less frequent use of benzodiazepines, and early physical and occupational therapy in the ICU are becoming established based on evidence generated from critical care trials, not only to reduce the duration of stay but also to minimise long-term cognitive sequelae^10)^. Outside of the ICU, formal cognitive rehabilitation, cognitive training, and psychosocial support interventions are being developed as potentially useful targets. Further interventional trials are required to confirm their effectiveness and determine timing and intensity.

The health effects worldwide are immense. Sepsis is still one of the most common causes of severe disease in many regions of Asia, Africa, and Latin America, but follow-up care is practically nonexistent. The awareness of post-ICU syndrome, including the cognitive aspect thereof, should be declared a public health concern in such areas. They are urgently required to implement cost-effective solutions, such as short cognitive screening instruments, which could be performed in the outpatient environment. Additionally, telemedicine-based post- discharge programs and caregiver-mediated cognitive tasks might become viable alternatives to professional neuropsychological services. Post-sepsis cognitive and psychological rehabilitation should also be incorporated in the fundamental healthcare packages by policymakers. The economic cost of untreated cognitive impairment in terms of lost productivity, dependence, and re-hospitalisation is perhaps even greater than the cost of early detection and rehabilitation.

The post-intensive care syndrome concept has justifiably broadened our knowledge of survivorship of critical illness. However, the cognitive aspect of such a syndrome is the most obscure and least discussed. Identifying it would necessitate a paradigm shift from short-term survival measures to long-term living standards. Future studies need to focus on the longitudinal follow-up of sepsis survivors, the pathophysiology of systemic inflammation combined with neurodegeneration, and the development of scalable cognitive interventions that can fit into diverse healthcare settings. Notably, clinicians need to start talking to patients and families about cognitive recovery openly, with realistic expectations and support resources.

To sum up, it is no longer about surviving sepsis. The psychological and cognitive impacts that remain can significantly alter the course of a survivor's life. As medical practitioners, we have a duty to go beyond preventing death to healing wholeness. The moment has come to close the divide between survival and recovery, to make sure that the minds we save are as sound as the bodies we repair.

## Author contributions

ZS conceptualized and drafted the commentary and approved the final manuscript.

## Conflicts of interest statement

The author declare that there are no conflicts of interest.
